# Reducing the Inadvertent Spread of Retracted Science: recommendations from the RISRS report

**DOI:** 10.1186/s41073-022-00125-x

**Published:** 2022-09-19

**Authors:** Jodi Schneider, Nathan D. Woods, Randi Proescholdt, the RISRS Team, Halle Burns, Katherine Howell, Mary Terese Campbell, Tzu-Kun Hsiao, Yee Yan ‘Vivien’ Yip, Yuanxi Fu, Yoss Arianlou

**Affiliations:** 1 School of Information Sciences, University of Illinois at Urbana-Champaign, 501 E. Daniel Street, MC-493, Champaign, IL 61820-6211 USA; 2 University of Lethbridge, Lethbridge, AB Canada; 3 Menlo College, Atherton, CA USA

**Keywords:** Retraction, Publication ethics, Citation, Spread of retracted research, Citation of retracted research

## Abstract

**Background:**

Retraction is a mechanism for alerting readers to unreliable material and other problems in the published scientific and scholarly record. Retracted publications generally remain visible and searchable, but the intention of retraction is to mark them as “removed” from the citable record of scholarship. However, in practice, some retracted articles continue to be treated by researchers and the public as valid content as they are often unaware of the retraction. Research over the past decade has identified a number of factors contributing to the unintentional spread of retracted research. The goal of the Reducing the Inadvertent Spread of Retracted Science: Shaping a Research and Implementation Agenda (RISRS) project was to develop an actionable agenda for reducing the inadvertent spread of retracted science. This included identifying how retraction status could be more thoroughly disseminated, and determining what actions are feasible and relevant for particular stakeholders who play a role in the distribution of knowledge.

**Methods:**

These recommendations were developed as part of a year-long process that included a scoping review of empirical literature and successive rounds of stakeholder consultation, culminating in a three-part online workshop that brought together a diverse body of 65 stakeholders in October–November 2020 to engage in collaborative problem solving and dialogue. Stakeholders held roles such as publishers, editors, researchers, librarians, standards developers, funding program officers, and technologists and worked for institutions such as universities, governmental agencies, funding organizations, publishing houses, libraries, standards organizations, and technology providers. Workshop discussions were seeded by materials derived from stakeholder interviews (N = 47) and short original discussion pieces contributed by stakeholders. The online workshop resulted in a set of recommendations to address the complexities of retracted research throughout the scholarly communications ecosystem.

**Results:**

The RISRS recommendations are: (1) Develop a systematic cross-industry approach to ensure the public availability of consistent, standardized, interoperable, and timely information about retractions; (2) Recommend a taxonomy of retraction categories/classifications and corresponding retraction metadata that can be adopted by all stakeholders; (3) Develop best practices for coordinating the retraction process to enable timely, fair, unbiased outcomes; and (4) Educate stakeholders about pre- and post-publication stewardship, including retraction and correction of the scholarly record.

**Conclusions:**

Our stakeholder engagement study led to 4 recommendations to address inadvertent citation of retracted research, and formation of a working group to develop the Communication of Retractions, Removals, and Expressions of Concern (CORREC) Recommended Practice. Further work will be needed to determine how well retractions are currently documented, how retraction of code and datasets impacts related publications, and to identify if retraction metadata (fails to) propagate. Outcomes of all this work should lead to ensuring retracted papers are never cited without awareness of the retraction, and that, in public fora outside of science, retracted papers are not treated as valid scientific outputs.

**Supplementary Information:**

The online version contains supplementary material available at 10.1186/s41073-022-00125-x.

## Background

  Retraction is a mechanism for alerting readers to unreliable material and other problems in the published scientific and scholarly record. Retracted publications generally remain visible and searchable, but the intention of retraction is to mark them as “removed” from the citable record of scholarship. As noted in the Committee on Publication Ethics (COPE) Retraction Guidelines, “The main purpose of retraction is to correct the literature and ensure its integrity” [1]. It may be applied to a number of problems (see Table 1). COPE also notes that “Prompt retraction should minimise the number of researchers who cite the erroneous work, act on its findings, or draw incorrect conclusions such as from ‘double counting’ redundant publications in meta-analyses or similar instances.” [1]. There are a variety of ways to view the purpose of retraction: alerting readers to unreliable material, cleaning up the literature, correcting the literature, amending the literature, etc. These phrases are used throughout this paper as different ways of describing retraction.

**Table 1:** Current retraction standards from the Committee on Publication Ethics (COPE) [1] and International Committee of Medical Journal Editors (ICMJE) [2]

	COPE	ICMJE
Reasons for retraction	• Unreliable findings • Plagiarism • Redundant publication • Lack of authorization to publish content • Legal issues • Unethical research • Published due to compromised peer review • Conflict of interest	• “Errors serious enough to invalidate a paper’s results and conclusions” ◦ Honest error ◦ Scientific misconduct • Duplicate publication
Retraction notice guidelines	• Link to retracted article • Clearly identify the article being retracted • Clearly identifiable as a retraction notice • Published promptly • Freely available • State who is retracting the article • State reason for retraction • Be objective and factual • Editors should negotiate with authors on wording	• Link to retracted article • Clearly identifiable as a retraction notice • Included in Table of Contents • Identify retracted article in heading • Include citation to retracted article • Authors of retraction notices should be authors of retracted paper when possible • State reason for retraction
Retraction process	• Editors should follow COPE Guidelines: Cooperation between research institutions and journals on research integrity cases [3] and CLUE guidelines [4] • Authors’ institutions should be notified of misconduct • Decision to retract ultimately falls to journals • If evidence of unreliability is inconclusive, retraction is usually not called for, but an expression of concern may be published • Retractions should not be issued solely because of authorship disputes	• Follow COPE guidelines when misconduct is alleged • Retract if misconduct is proven; if inconclusive, letters to the editor may be published to show areas of debate • Previous works of the author of a retracted paper should not be presumed invalid, but expressions of concern may be published
Citing retracted papers	Not covered by COPE Retraction Guidelines	• References should be checked using an electronic bibliographic source, or an original print source • Authors should be sure not to cite retracted papers without acknowledging the retraction • PubMed is an authoritative source for retraction information

The spread of retracted science can lead to real harms in clinical or other settings. For instance, in 2003, a study was published that reported two medications were more effective in combination than alone [5, 6]. The study was retracted six and a half years later, after over 100,000 patients had been given this treatment, exposing them to dangerous side effects [6]. Additionally, R. Grant Steen found that 17,783 patients were put at risk by 180 different retracted clinical studies in PubMed, and 165,588 were put at risk by other studies that cited these retracted studies [5]. Retracted research can pose a threat to patient health as well as misdirect time, energy, and funding of those involved, sometimes at the expense of taxpayers [5, 6].

Another well-known case is the 1998 Wakefield et al. study that linked the MMR vaccine to autism. The study was fully retracted in 2010 due to lack of ethical approval, flawed study design, and “incorrect” elements [7], but has continued to receive citations in recent years. The majority of these citing papers disagree with the Wakefield paper and/or acknowledge its retraction [8, 9], perhaps in part due to the amount of attention its retraction received. However, despite its retraction, the paper may have had a negative impact on public opinion of the MMR vaccine [10], as well as vaccination rates, e.g. in Britain [11].

The spread of retracted research has also played a role in the misinformation surrounding the COVID-19 pandemic. Over 200 articles related to COVID-19 have been retracted during the first two years of the pandemic [12], yet many of them continue to be cited according to early studies. As of fall 2020, 33 retracted papers had already been cited by 236 other papers; the papers that cited those papers (“the second generation of polluted science”) had been cited 834 times [13]. Frampton et al. analyzed the first 46 COVID-19-related retractions, and found that more than half could be found in their original form (i.e., with no mention of the retraction) on a variety of websites, as of December 2020 up to 8 months after the retraction [14].

In addition to its clinical impact, the spread of retracted science can affect how people interact with the environment and wildlife around them. For example, a study published in *The Proceedings of the National Academy of Sciences (PNAS)* found that closing 5% of the ocean to fishing would improve catch rates by 20%, but was retracted due to errors and possible conflict of interest [15]. The study remains cited in the United States Congressional Record as supporting documentation for a bill proposed in November 2020 [16]. Another study, published in 2009 and retracted in 2012 due to unreproducible results, reported that antibiotics in the carcasses of livestock had negative health effects on vultures. Supplementing vulture diets with dead livestock is a common practice, and the paper’s findings called this practice into question. Removing livestock carrion would have been a reduction in vultures’ food supply, and could have resulted in a decline in vulture populations, including the Egyptian vulture [17]. This species is endangered, with numbers in rapid decline due to multiple factors including the drug diclofenac [18], a different veterinary drug than the one that the retracted paper had alleged to cause harm to vultures. Unreliable information presented by the retracted paper could have complicated efforts to identify the factors in the Egyptian vulture’s decline and to prevent further population loss. These examples demonstrate the potential harms of retracted research and the need to prevent it from spreading.

Research over the past decade has identified a number of factors contributing to the unintentional spread of retracted research. Many retracted papers are not marked as retracted on publisher and aggregator sites [19, 20], and retracted articles may still be found in readers’ electronic libraries, including in reference management systems such as Zotero, EndNote, and Mendeley [21]. Seventy-five percent (75%) of the retracted papers investigated were available in personal Mendeley libraries as of 2012 [21], and readership of retracted papers on Mendeley continues to grow after retraction [22]. Further, electronic copies persist beyond the publisher site [21], and large numbers of sites may redistribute the same paper, without indicating its retraction. It is unknown how many publishers systematically surveil bibliographies of submitted manuscripts, or how many editors query whether a citation to a retracted paper is justified. While relevant technology exists (e.g., [23]), it is limited by metadata quality.

When citing retracted papers, authors frequently do not indicate retraction status in bibliographies or in-text citations. A study of citations to retracted papers in PubMed found that only 5.4% of post-retraction citations acknowledged that the paper they were citing was retracted [24]. A smaller study, focused on two retracted COVID-19 articles, found that 52.5% of citations did not acknowledge the fact that they were retracted despite the widespread media attention that these retractions received [25]. In addition to spreading information without notifying readers that the source was retracted, the citation of retracted papers can also impact the validity of the paper that is citing it, including meta-analyses that include retracted papers [26].

While some databases have indexing terms for retracted publications, in practice, indexed data is incomplete. For example, an analysis of PubMed's duplicate publication index in 2013 found 25 retracted publications (identified by publisher notices), many of which (12; 48%) were not in the retracted publication index; these problems persisted after contacting PubMed and editors during a 5-year followup period [27]. A 2020 search found that retraction document types were missing for 58 items in PubMed, 56 in Web of Science, and 8654 in Scopus (the majority of items in Scopus have since been corrected); this study identified whether items were retracted based on retraction labeling in the article title, so there were likely additional articles it didn’t identify [28]. A study of the mental health literature found that only 60% of database records of retracted items indicated retracted status, and of those that did, the majority only indicated the retraction in one place [29]. Another study discovered indexing issues in both document titles and the linking of retracted publications and retraction notices [30]. Such data quality problems may persist for years, despite efforts to alert journal editors and databases.

Multiple suggestions have been made for ways to reduce the citation of retracted articles (e.g., [31]). Crossmark [32, 33] was developed in part to enable readers to check that they have the most up-to-date version of an article. Indicating retraction through watermarking PDFs and flagging retraction on HTML pages is important; non-watermarked retracted articles are more cited than watermarked ones [34], but not all retracted articles are watermarked [14, 35]. Prepending titles with "RETRACTED:" in databases and scholarly search engines has also been suggested. Systems to surveil bibliographies in pre-press manuscripts have been suggested numerous times (e.g., [21, 36]). Researchers in diverse fields have called for reference management systems to flag retracted articles [37, 38], and currently we are aware of three that do, using data from Retraction Watch: Zotero since June 2019 [39]; Digital Science's Papers since September 2021 [40]; and EndNote since November 2021 [41]. Another common recommendation has been to remove retracted papers entirely (e.g., [22, 36, 42, 43]); for instance, Rzymski suggests “hard retraction” to handle “cases of fraud or grave errors with broad impacts,” as a mechanism for removing articles from the publisher site and most indexes, except for a limited access repository dedicated to retracted papers [43]. Alerting authors who previously cited newly retracted work has also been suggested [44, 45].

Following established guidelines can also help reduce citation of retracted papers. However, practices and norms vary by field. Table 1 shows COPE and ICMJE retraction guidelines. ICMJE is focused on medical journals, and, while COPE has since become interdisciplinary, it was originally created by biomedical journal editors [46], and some of its early guidelines were explicitly focused on the biomedical field [47]. Much of the existing empirical research about retraction is also focused on biomedical fields, perhaps due in part to factors such as the high stakes involved with clinical research and the freely available data in PubMed [48]. However, retraction can occur in any field, though reasons, context, and rates may vary. For example, in the arts and humanities, the majority of retractions are related to recycled work and plagiarism, and a low proportion is related to issues related to data errors [49]. One study found that retractions in the engineering field were also primarily due to plagiarism and self-plagiarism [50], though another study found plagiarism to be second to unethical research as the most common reason for retraction in engineering [51].

Some studies have found that rates of retraction are higher in certain fields, such as medicine, chemistry and non-medical life sciences [52]. However, it is important to note that greater rates of retraction do not necessarily indicate greater rates of error, falsification, or fraud in a particular field. A greater number of retractions can instead indicate greater attention to scientific integrity and validity, as the scholarly community devotes greater attention to removing errors and fraud from the literature [53, 54]. In particular, applied fields may gain scrutiny from a wider audience than more abstract ones, and the extent to which work is empirically grounded in evidence that is directly reported can vary between fields.

The goal of the Reducing the Inadvertent Spread of Retracted Science: Shaping a Research and Implementation Agenda (RISRS) project was to develop an actionable agenda for reducing the inadvertent spread of retracted science. This included identifying how retraction status could be more thoroughly disseminated, and determining what actions are feasible and relevant for particular stakeholders who play a role in the distribution of knowledge, including researchers, authors, editors, librarians, data and metadata providers, research integrity organizations, and standards organizations.

## Methods

To derive recommendations, we used a year-long process. Briefly, this included a scoping review of empirical literature (See Appendix C: Literature Scoping Review Methods and Intermediate Results in [48] and the online bibliography [55]) and successive rounds of stakeholder consultation (See [56]; further details are in the Appendix D: Stakeholder Consultation Process in [48]; workshop attendees are also listed on the project website [57]). The project culminated in a three-part online workshop (October 26, November 9, and November 16, 2020) seeded by materials derived from stakeholder interviews held May 2020 through November 2020 and short original discussion pieces contributed by stakeholders. After the workshop, stakeholders were involved in several rounds of direct feedback to iterate the recommendations before the final RISRS report [48].

Our process was loosely premised upon a five stage participatory agenda setting model [58, 59] involving exploration, engagement, prioritization, integration, and dissemination. The exploration phase consisted of preliminary literature review and the formation of a stakeholder advisory board.

Sixty-five stakeholders were formally enrolled (9 stakeholders participated just in the interview process, 18 in the workshop alone, and 38 participated in both interview and workshop). 23 stakeholders participated from the research enterprise (e.g., researcher, research support, research integrity); 18 from publishing (e.g., editor, publisher, publishing policy, publishing process); 12 from infrastructure (e.g., software, platforms, communication, metadata); and 12 from policy and standards (e.g., funder, government official, policy commenter, journalist, standards organization). For these counts, we treated 2 group interviews with a total of 6 interviewees as 2 stakeholders.

Interviewed stakeholders were asked to discuss their knowledge and experience with the retraction process, opinions regarding retraction, and strategies for mitigating the continued circulation of retracted materials. In the prioritization phase of the cycle, the qualitative analysis derived from the consultations and preliminary literature reviews were initially presented to stakeholders for feedback in Workshop 1 described below. Workshop 2 and 3 initiated the integration phase, where stakeholders were asked to refine and prioritize problems and opportunities [see also Additional file 1].

To prepare the initial agenda for the workshop, the RISRS team (NDW) thematically analyzed interview transcripts and quotations about problems and opportunities extracted as part of the literature review. Thematic analysis [60] is a means to organize and describe patterns within data in rich detail, and was chosen to help facilitate sensemaking activities with stakeholders [60, 61]. First, interview transcripts were coded inductively using in vivo codes, or codes derived from the data, to identify patterns of meaning or relevance attributed by the interviewees to the retraction process [62]. This initial coding produced 160 in vivo codes, which were constructed and iteratively modified as interviews were completed. They were subsequently categorized into higher order concept codes (78 in total).

In parallel to the interview coding, articles from the preliminary literature review were coded using the above strategy, moving from inductive in vivo codes, to deductive coding of problems and opportunities. Interview and document coding resulted in 41 distinct codes associated with problems and 38 distinct codes associated with opportunities for addressing the problem of retracted research in the scholarly communications ecosystem [63].

These analytic codes were then used as the basis for the development of a set of 6 preliminary themes, or a conceptual description that named discrete groups of patterns pertaining to the broad meaning of retractions. These themes were written up using illustrative quotes that exemplified elements of the themes. This document was circulated prior to the workshop as a type of ‘member check,’ or respondent validation, where stakeholders checked the narrative accuracy, and interpretive and descriptive validity of the quotes as paired with the themes.

Each workshop session was developed around a particular group task, drawing on modified problem structuring approach [64]: Workshop 1 focused on listening and learning about stakeholder experience with retraction from a variety of participants; Workshop 2 on collaborative agenda setting, where stakeholders prioritized problems and opportunities; and Workshop 3 on implementation topics, such as barriers to cooperation, and sustaining commitment to act in the short- and long-term. Topics selected on the basis of the qualitative analysis were discussed using structured conversation patterns from Liberating Structures [65], encouraging participants to collaborate to develop solutions, reframe proposed pathways to reform, and to nominate alternative framings or problem sets. Shared documents from each workshop were subsequently analyzed by the RISRS team (NDW), along with notes from the RISRS team's (HB, JS, MC, NDW, RP, TH, YA, YF) embedded participant observations, utilizing the coding strategy outlined above.

The RISRS team produced a series of four drafts over a period of six months, with one internal and two public [66, 67] drafts before the final report [48]. Major problems and opportunities identified through the literature review, interview, workshop and survey process were described in the first, internal draft. The interview was presented to stakeholders about a week before a short working meeting (February 16, 2021) held to gauge stakeholder perceptions, and to give space for discussion for additional suggestions or refinements. Stakeholders contributed to these drafts by reviewing wording, concept formulation and supporting inferences. This iterative and ongoing feedback process helped to scope recommendations from broad concerns, to clear possibilities for action. Public drafts also received feedback from a broader community. Following comment and feedback, the recommendations stabilized and additional background and a research agenda and implementation model were added to the recommendations document.

### RISRS Project Assumptions

Stakeholder-engaged research is co-constructed between the researchers and the stakeholders: since the research involves human interaction, the researchers are an “integral part of the research design, data collection and the research outcomes” [68]. Our primary perspective drew on the project director JS's ongoing research in scholarly communication since 2006, and the lead qualitative researcher NDW's expertise in science policy and applied anthropology. Members of the RISRS team who participated in the workshop as notetakers and facilitators (HB, JS, MC, NDW, RP, TH, YA, YF) contributed a multidisciplinary background including graduate training in anthropology: NDW; bioinformatics: YF; chemistry: YF; finance MBA: MTC; library and information science: HB, JS, NDW, RP, TK; math: JS; informatics: JS; or undergraduate training in history and informatics with experience in interlibrary loan support: YA. While we count the start of the project from its funding in February 2020, JS's initial views were shaped, starting in 2017, from reading the scientometrics and scholarly communication literature published since 1990 about the problems of citation to retracted articles and multiple solutions. Scientists' voices were amplified by this literature, but the voices of editors and publishers were more rare.

Given the repeated attention to citation of retracted papers since the 1990's, [13, 21, 69–75], we knew that the inadvertent spread of retracted science was a complicated and long-standing issue. We started from the assumption that this was a “stuck” problem that would require collaboration across diverse stakeholders and, possibly, systemic changes to the way people work across scholarly communication.

We conceptualize scholarly communications as an ecosystem comprising the individuals, institutions, and processes through which research is produced, packaged, managed, disseminated, promoted, consumed, and preserved into something deemed the scholarly record. Examples of individuals include publishers, editors, researchers, librarians, standards developers, funding program officers, and technologists. Examples of institutions include universities, governmental agencies, funding organizations, publishing houses, libraries, standards organizations, and technology providers (e.g., which include vendors providing search engines, databases, abstracting and indexing services, computing platforms and other infrastructure, citation software, etc.). Examples of processes include: submitting, peer reviewing, or accepting a manuscript; quality assurance; typesetting; creating metadata; depositing data; curating research products; selling, licensing, and acquiring books, journals, etc. These are embedded in material, social, and technical processes, which we conceptualized as points of intervention. By working with stakeholders from across the ecosystem, we endeavored to understand how people interact with retracted research, and encouraged stakeholders to reflect on how these interactions could be redesigned to interrupt the continued citation of retracted research in these chains of research communications.

### Scope

Our scope is primarily, but not exclusively, the continued citation and use of retracted research. Our investigation at times strayed beyond *citation* and *use* alone. That is because, for many of the sources and people we consulted, citation and use are intertwined with a much broader frame of reference, including the goals, purpose, and meaning of retraction, expectations regarding its implementation, and other aspects.

We adjusted our scope as we consulted with stakeholders and learned about new perspectives on the citation and use of retracted research, as well as areas of disagreement. Part of the work of this project has been to move from the widespread view of retraction as a social problem to a common stakeholder agenda specifying the range of problems associated with retracted research and its continued citation. Collaboration across major stakeholder groups is challenged by lack of common agreement about the scope of the problem, or the efficacy of strategies to address the issue. For example, retracted research is sometimes framed as an issue of individual misconduct or accountability. Likewise the continued citation of retracted research may be framed in terms of breakdowns in editing and publishing processes. Such differences in aim and perspective lead to some tension about what is needed, and what is possible. On the one hand, many interviewees called for substantive reform of the scientific publishing ecosystem itself and its role in scientific careers. On the other hand, other interviewees called for fine-tuning of current practices and processes in effect to optimize the retraction process, and clarify the role of retraction in stewarding the scientific record. This unresolved tension has limited efforts to build the will and capacity to address the continued spread of retracted research.

## Results: the Recommendations

By using the process outlined above, we developed 4 broad recommendations (Table 2). Rather than target a particular sector, or problem, these recommendations speak to multiple points in the scholarly communications ecosystem.

**Table 2:** Reducing the Inadvertent Spread of Retracted Science (RISRS) Recommendations

	Publishers/Journals	Standards organizations	Researchers	Other stakeholders
**Develop a systematic cross-industry approach to ensure the public availability of consistent, standardized, interoperable, and timely information about retractions**	(1) Adopt standards for citation of retracted papers, and for labeling retracted papers, (2) use software solutions, (3) invest in metadata quality	Develop standards for (1) retraction labeling in databases and publisher websites, (2) best practices for availability of retraction information in databases	Use citation software to flag retractions	Citation software developers: develop flags for retracted papers
**Recommend a taxonomy of retraction categories/classifications and corresponding retraction metadata that can be adopted by all stakeholders**	Adopt, and participate in developing, a retraction taxonomy	Develop and maintain retraction taxonomy	Become aware of taxonomy and pay attention to classifications of articles being cited	Working group composed of a variety of stakeholders: develop retraction taxonomy
**Develop best practices for coordinating the retraction process to enable timely, fair, and unbiased outcomes**	Reserve the right to retract in legal agreements; provide clear instructions for inquiries/concerns	Clarify best practices	Follow CLUE report recommendations	Funders, research institutions: follow CLUE report recommendations; Research integrity organizations: clarify best practices
**Educate stakeholders about pre- and post-publication stewardship, including retraction and correction of the scholarly record**	Develop education aimed at multiple groups; build awareness of existing resources	Develop best practices for emerging concerns such as preprints; support authors in identifying authoritative sources for checking citations	Evaluate and assess references; clearly indicate if cited work is retracted; notify publisher if cited work in evidence synthesis is retracted; notify publisher, institution, and coauthors of issues with a published article	Scholarly societies, government agencies, and local institutional programming: develop education aimed at multiple groups

### Develop a Systematic Cross-industry Approach to Ens﻿ure the Public Availability of Consistent, Standardized, Interoperable, and Timely Information about Retractions

Over 94% of post-retraction citations in biomedicine do not demonstrate awareness that the cited item was retracted [24]. Users' typical citation workflows may involve citing preprints, reusing downloaded copies, citing older works contained in their reference managers, and copying citations from their own or others' previous bibliographies [21, 22]. Among citation styles, only the American Medical Association [76], National Library of Medicine [77], and American Psychological Association [78] styles provide explicit standards for citing retracted papers (See Appendix E: Existing Citation Standards for Retracted Publications in [48]). Among commonly used systems, only a handful of databases (such as PubMed and RetractionWatch) and tools built on them (such as Zotero, EndNote, Papers and scite) ensure that users know that a paper they are citing is retracted.

Information about retraction needs to move across different industry information providers (publishers, abstracting and indexing services, scholarly search engines, etc.). However, currently this need is challenged by non-robust dissemination, inconsistent information, and inconsistent presentation of retraction status information [30, 79–81].

Shared standards amongst publishers are necessary, but currently there are no industry-wide standards for retraction information or its visibility. The most widely accepted guidelines, from the Committee on Publication Ethics (COPE) [1] and the International Committee of Medical Journal Editors (ICMJE) [2], recommend how to make retraction information easy to use and find. However, they are not uniformly adopted. Although both are widely accepted by many publishing groups, particularly in medicine [82, 83], previous research has found that publishers do not uniformly adhere to COPE and ICMJE recommendations [84–86] and that more consistent display standards are needed, particularly regarding uniformity in landing pages [81]. In 2015, Retraction Watch also published its own standard for what a retraction notice should include, with more details than those of COPE and ICMJE [87].

Supporting and motivating stakeholders to consistently adopt and follow COPE and ICMJE recommendations for managing retracted articles and retraction notices is a baseline for further improvements. Beyond COPE and ICMJE recommendations, publishers should update procedures to add ‘Retracted’ to the titles of retracted articles following the example of the database Web of Science [27, 88].

A standards group should develop best practices for databases that facilitate the public and unrestricted access to and dissemination of retraction statuses and retraction notices. Processes, model license agreements, and standards for retraction data interchange are needed to facilitate information flow between publishers, aggregators, and database providers. Model license agreements could expand on established agreements such as the National Library of Medicine's participation agreement for deposit [89].

Future models for disseminating retraction status include specialized databases such as the Retraction Watch Database, inclusion in field-specific databases such as PubMed, and/or as metadata in centralized repositories such as DOI registrars including CrossRef and DataCite and others. These data sources are not mutually exclusive, and ideally, retraction status would be up-to-date in all sites where readers encounter publications. Although a well-curated infrastructure could, in principle, be developed from centralizing metadata sourced from publishers, in practice, relying solely on publishers poses challenges because different publishers show varied and sometimes inadequate resources and commitment to metadata maintenance (see, for instance, the metadata improvement efforts of Metadata 20/20 [90]). General and field-specific databases also currently curate retraction metadata, but again, the quality varies [28]. Centralized metadata maintained by an external group focused solely on retraction, such as the Retraction Watch Database, ensures high quality, however, it requires an ongoing commitment, with financial resources for skilled curators and technological infrastructure.

Sustainable funding sources are urgently needed for databases to facilitate the public and unrestricted access to and dissemination of retraction notices. For example, the difference between restricted access and unrestricted access, facilitated by funding, can be seen by comparing the Retraction Watch Database to PubMed. Retraction Watch is a public database with restricted access to over 32,000 retractions in all disciplines [91]. Free public results are limited to 600 and license agreements are required for bulk use, and (as of January 2022) there is no Application Programming Interface (API). Its funding has included grants, private donations, and licensing agreements. PubMed is a public database with unrestricted access to and dissemination of over 10,000 retraction notices [92] in biomedicine. PubMed's public interface and API are free to users because it is completely funded by the United States government. For other databases, such as Retraction Watch, that are not government-funded, additional funding sources could help make retraction information more free and accessible, especially through automated electronic means of data retrieval (e.g., APIs) to track and disseminate retraction statuses.

Another working group should convene composed of reference and citation industry groups along with members from COPE, the National Information Standards Organization (NISO), and others. The working group should be charged with defining best practices addressing retraction and post publication amendments in citation styles and citation software; developing additional citation styles and standards for indicating the retraction or correction status of a paper in text and in a bibliography.

Multiple stakeholders can play a part in adoption. Citation software developers should add features to flag retracted papers in their tools (e.g., Mendeley, Paperpile, RefWorks, etc.); Zotero, which flags retracted papers based on DOIs in Retraction Watch Database data, can be used as a model, as well as EndNote [93] and Papers [40], which announced the integration of Retraction Watch data in Fall 2021. Researchers should use citation software that flags retracted papers. Submission management platforms should integrate tools that enable systematic identification of retracted articles. Publishers should adopt software solutions that enable systematic identification of retracted articles in bibliographies prior to publication and check bibliographies for retracted paper as part of manuscript review and publishing workflows. Publishers should also invest in maintaining metadata, including promptly registering post-publication amendments with Crossmark [33], which became free to Crossref members in March 2020 [94].

### Recommend a Taxonomy of Retraction Categories/Classifications and Corresponding Retraction Metadata that can be Adopted by All Stakeholders

A COPE working group noted in 2017 that “No standard taxonomy of updates exists for publishers to adopt. This leads to inconsistencies from journal to journal and potential confusion for the reader” [95]. Currently, retraction notices often provide vague or limited information about the reasons for retraction [84, 96, 97]. Terms currently in use are not used consistently: for instance, “withdrawal” is currently used in different ways [98–100].

People using retracted science and evaluating authors of retracted science demand additional context about retraction to both clean up the literature and disincentivize misconduct [101]. For example, researchers concerned with the stigma of retraction would like to distinguish retraction due to honest error from retraction due to misconduct [102, 103]. Some journals use “retract and republish” [104] or “retract and replace” [105] to signal handling of ‘honest’ errors; but some journals “keep the same DOI for the original and retracted article”, leading to poor indexing of these items' retraction statuses in bibliographic databases [106].

Several taxonomies have been suggested in the literature: Fanelli et al. proposed definitions for 13 types of amendments, differentiated by asking: What is the issue? What is the impact? Who caused it? and Who communicated it? [102]. A bottom-up classification of retraction notices published in the journal *Science* led Andersen and Wray to 12 categories of error (4 levels of impact × 3 characterizations of intentionality) [107]. However, concerns about possible reputational damage and the risk of litigation can disincentivize the use of fine-grained distinctions about reasons for retraction [108]. A 2017 COPE working group advocated simplicity, using identifiers to interlink publicly available documents [95] but reducing the complexity of the classification. Their proposal is centered around ‘use of the neutral term “amendment” to describe all forms of post-publication change to an article’ [95]; each amendment notice would indicate who was issuing it and any dissenters; the type (“minor”, “major”, or “complete”); links to the article being amended and associated resources; the date; and an associated narrative, that is “updated as needed with links to any investigation if that is publicly available” [95]. More recently, a 2021 COPE RISRS taxonomy working group made an initial proposal to coalesce on 5 or 6 essential terms [109].

We recommend that a working group composed of standards organizations, publishers, platforms, infrastructure providers, and metadata development organizations work to develop a core taxonomy of retraction categories and corresponding metadata standards in tandem. The corresponding metadata standards should draw on existing models of persistent identifiers, versioning, and explicit links between expressions of concern, retraction notices, and the publications to which they refer. These links should be both machine-actionable and human-understandable. The taxonomy should be integrated with existing versioning systems. The working group should recommend how the taxonomy's terminology should appear in database records for retraction notices and retracted articles when the taxonomy is implemented and adopted.

Models of persistent identifier usage may come from best practices for research outputs beyond the traditional scholarly journal article, including preprints, data, software, study protocols, registered reports, and repository content. Examples of best practices include (1) resolving a persistent identifier to a tombstone page [110] when the full-text must be removed; (2) providing versioned DOIs (e.g. Zenodo's versioned DOIs for software [111]) so that substantive changes to the content of an item are reflected by a change to the persistent identifier; and (3) using explicit metadata to interlink versions (e.g., DataCite Metadata Schema [112]'s metadata for recording relations such as "IsNewVersionOf" and "Obsoletes" in DataCite Metadata Schema [112], or more general Crossref intra-work relationships such as "isReplacedBy" and "Replaces" [113], as well as older best practices for publications (e.g., the 2008 Journal Article Versions (JAV): Recommendations of the NISO/ALPSP JAV Technical Working Group [114])).

Within contemporary scholarly article publishing, F1000 provides an example of explicit versioning with the use of persistent identifiers: "All versions of an article are accessible, each with their own DOI (digital object identifier) and may be cited individually." [115]. Their website has a useful interface for ensuring that human readers are alerted to the most recent version of an article. For a reader browsing an older version of an article, the F1000 website provides a daily notification which states: "There is a newer version of this article available." Similar notifications exist on preprint servers and data repositories to indicate new versions, typically in banner messages. Yet at F1000 the content cannot be viewed before clicking on "Suppress this message for one day": this interaction design ensures that a human reader with standard web browser settings cannot miss the message. Adoption of similar alerting would address one the most challenging current problems: ensuring that readers are notified about retraction.

In order to ensure the taxonomy and metadata are viable over the long-term, they should be curated and maintained on a discoverable website with a formal home and be based in an industry standards organization such as the NISO or the International Association of Scientific, Technical and Medical Publishers (STM). Finally, to ensure adoption, we recommend that highly visible organizations build support and influence through endorsement and adoption of the taxonomy and metadata standards, in order to support and motivate other stakeholders to adopt them.

### Develop Best Practices for Coordinating the Retraction Process to Enable Timely, Fair, and Unbiased Outcomes

The time between the publication of papers and their potential amendment or retraction is a period in which papers may be adopted, used, and woven into the tapestry of scholarship. The need for retraction can be raised at any time after publication, and this time has been as long as 45 years (e.g., [116]). From the point of view of citation and use, there are two interrelated issues: First, the longer a publication is "alive" in the literature before retraction, the more time it has had to accrue citation and use while considered normal citable literature; this increases the potential impact on the rest of the literature, because there is currently no systematic process for updating knowledge claims when publications have already been cited by the time they are retracted [45]. Second, publications with shorter time to retraction may also receive fewer post-retraction citations [24]. Reducing the time to retraction is desirable to ensure the clear and timely communication of amendments to publications.

Another danger is that compromised research is identified but fails to be retracted because of logistical complexity amongst all stakeholders involved in the retraction process. Additional complications may arise when the author or editor is no longer publishing or no longer living [117, 118]. In these cases, failure to retract enables the continued citation of research that should have been retracted. Likewise, transfer of journals between publishers may adversely impact the display of retraction status.

Existing guidelines acknowledge the problems related to time to retraction. For example, the COPE 2019 guidelines say: “Publications should be retracted as soon as possible after the editor is convinced that the publication is seriously flawed, misleading, or falls into any of the categories described above.” However, stakeholders suggest that coordination amongst authors, co-authors, editors, and in some cases institutions may present complex logistical problems or conflicts of interest. For example, review of compromised figures, data sets, and data represented in images can be costly and time consuming. For editors and publishers, the COPE flowchart library is in common use, and could be a model for developing workflow models and suggestions aimed at a variety of additional stakeholders. Some interviewees and workshop participants suggested that efforts to innovate retraction processes in this nexus—between institutions, publishers/editors, funders and researchers/editors—are often hampered by perceptions of risk and liability. Early adopters of reforms potentially face increased risks (e.g., liability) on top of the cost of developing policies and procedures; potential costs include referral boards or independent investigative bodies.

Here, we recommend the use of the Cooperation & Liaison between Universities & Editors (CLUE) report [4] recommendations to develop best practice guidelines to streamline the retraction process with respect to institutions and sponsoring agencies by improving coordination between institutions, publishers, funders and researchers. Additionally, COPE and the research integrity groups such as the Association of Research Integrity Officers (ARIO) and the European Network of Research Integrity Offices (ENRIO), should work to clarify best practices and guidelines for journals, authors, and institutions to efficiently coordinate and address concerns about published work. This should include offering fast-tracks for retraction notices to move through the process more quickly, if the authors agree with or request retraction, or if a retraction is requested following an institutional misconduct investigation. Here, publishers should reserve the right to retract in legal agreements with authors. Publishers should also make sure that all journal websites provide clear instructions on how to submit an inquiry or concern about possible research misconduct or serious error. For instance, websites may not have updated contact information or email addresses. Finally we suggest creating a workflow template for starting a retraction inquiry and adopt a checklist of requisite information for a retraction notice. Publishers and editorial societies should encourage journal editors and institutions to develop systematic processes, including templates and checklists to coordinate and communicate about the retraction inquiry.

### Educate Stakeholders about Pre- and Post-publication Stewardship, including Retraction and Correction of the Scholarly Record

Stakeholder education can help researchers and editors understand the range of post-publication corrections. Retraction is a publishing mechanism for cleaning up the literature, and does not signify misconduct. Currently stakeholders report a tension between the need to correct the literature and the need to preserve their reputations, either as researchers or as editors. Fear of stigma or career impacts can make researchers reluctant to participate in retraction processes, even to correct honest mistakes or errors. Fear of litigation makes editors reluctant to initiate retraction inquiries [1, 119]. Awareness of retraction and the reasons for retracting research may vary by field; this contributes to a confusion about the severity and impacts of retraction. Professional, disciplinary and scholarly societies, publishing associations and editorial groups, government agencies, and local institutional programming should develop education aimed at multiple groups. Our detailed recommendations for researchers, authors and editors can be found in the RISRS report [48]. These are examples; education for additional stakeholder groups, for example, librarians, developers of bibliographic databases and search engines, and research integrity officers, should be developed. Scholarly publications are used not only within the communities that produce them, but also more widely for application to public decisions: in the future, ​​science communicators and journalists as well other knowledge brokers who help the public interpret scholarly communication could be a target for further education as well.

## Discussion

The inadvertent citation of retracted research is a long-standing and severe problem. Our stakeholder engagement study led to 4 recommendations to address this problem: timely and consistent information display; a taxonomy and metadata to clarify core retraction statuses; best practices for coordinating the retraction process; and stakeholder education to reduce stigma and increase acceptability of the full range of post-publication corrections. Many of these recommendations resonate with the independent, pre-existing suggestions of authors, editors, and publishers in the prior research. The advantage of a stakeholder consultation process is that it builds consensus on priorities and momentum towards change.

To implement these recommendations, standards development is the next logical step, in order to build further consensus, and to develop actionable implementations that can be piloted. As a result of the RISRS stakeholder engagement, a NISO working group is developing a Communication of Retractions, Removals, and Expressions of Concern (CORREC) Recommended Practice, starting in May 2022 [120, 121]. This work will focus on the first two recommendations: display issues and metadata for retraction notices, expressions of concern, and related documents. The recommended practice will ensure consistent, timely dissemination of retraction information to both machine and human readers, directly or through citing publications.

To be successful, implementation of the recommendations will need financial resources from public and private funders as well as in-kind resources from publishers, metadata and technology infrastructure organizations. Compared to the RISRS project, which was limited by language barriers, timezone coordination, and interpersonal networks to a largely North American and Western European stakeholder group, standards development should seek wider and more representative participation.

In the near term, additional research should also be funded to support standards development. Research can address practical questions, such as 1) How well is retraction currently documented within multidisciplinary databases and search engines? 2) How does retraction of code and datasets impact related publications? 3) What are some success and failure use cases for retraction metadata, showing how retraction metadata successfully propagates, or fails to propagate? The research questions are chosen because of their ability to support near-term action. The first question, for example, could provide data-driven evidence on the scope of the current metadata indexing problem. We believe this could help secure the sustainable funding sources that are urgently needed for databases to facilitate the public and unrestricted access to and dissemination of retraction notices. The second question is timely given the increasing attention to scholarly objects, beyond publication, even in the space of data ethics: in September 2021 a new COPE/FORCE11 Research Data Publication Ethics taskforce published their best practice recommendations [122, 123]. The third question will develop hypotheses about what is going right and wrong, which will help design better metadata workflows in the future.

The NISO CORREC standards development process is the first stage of a multi-step implementation strategy; once the Recommended Practice is developed, it will need pilot testing, followed by wider dissemination once it is validated. Further research will also be needed in the future. These particular actions—standards development, and research specifically supporting it—are the highest priority in the short-term from the more detailed implementation strategy and research agenda presented in the full RISRS report [48].

### Limitations

Before consulting stakeholders, we began this project with assumptions about what the problem was and how to address it. Our underlying assumption developed primarily from reading the existing published literature about retraction, and from experience in librarianship, scholarly communications research, and related areas. In particular, we assumed that a collaborative effort among various stakeholders would be necessary due to the limited progress made over the 30 years scientometrics researchers had been attending to retraction. As we began to involve stakeholders, we may have influenced them with our preconceived notions and our understanding of the problem.

## Conclusions

Addressing the inadvertent, continued citation of retracted science will require iterative work from many parts of the scholarly communications ecosystem. Early implementation activity coming out of the RISRS process has resulted in some promising initiatives that look at how to develop these recommendations in ways that will meaningfully address long-standing issues in the scholarly communications ecosystem that contribute to the continued citation of retracted materials. The COPE taxonomy working group and the proposed NISO work item are helping to address this issue by advocating for stakeholders to address the interlinked issues, but also innovating practical solutions to be further co-developed in specific sectors of the scholarly communications enterprise.

Yet there is still more work to be done, and we hope that by synthesizing this material we not only further the conversation about how to best address the problems presented by the continued circulation of retracted research, but as well support stakeholders in developing practical and actionable strategies within their zone of influence. It is our hope that further working groups take up the recommendations outlined above, as well as some of the targeted implementation priorities that we highlight. We welcome suggestions for ongoing research and collaboration, via the project website https://infoqualitylab.org/projects/risrs2020/ or by email to jodi@illinois.edu.

## Supplementary Information

**Additional file 1. **Online Supplement Description: Further description of RISRS workshops and excerpts from 2 surveys of workshop participants.

## Data Availability

The full RISRS report informing this publication is available on MetaArXiv Preprints [48]. Many project outcomes are in IDEALS, the UIUC institutional repository, in the RISRS Collection https://www.ideals.illinois.edu/handle/2142/108359 and on the RISRS project website https://infoqualitylab.org/projects/risrs2020/. Data: We have deposited the qualitative analysis of materials used in the RISRS report in the UIUC Databank [63]. Given the risk of identification, we are unable to share full transcripts of the interview data.

## Abbreviations

ARIO: Association of Research Integrity Officers
COPE: Committee on Publication Ethics
ENRIO: European Network of Research Integrity Offices
ICMJE: International Committee of Medical Journal Editors
NISO: National Information Standards Organization
RISRS: Reducing the Inadvertent Spread of Retracted Science
STM: International Association of Scientific, Technical and Medical Publishers
