# Supplementary material for: Reducing the Inadvertent Spread of Retracted Science: recommendations from the RISRS report
Source: Res Integr Peer Rev. 2022 Sep 19;7:6. doi: 10.1186/s41073-022-00125-x (PMC9483880; doi:10.1186/s41073-022-00125-x)
Supplement: Supplementary file 1 — Additional file 1. Online Supplement Description: Further description of RISRS workshops and excerpts from 2 surveys of workshop participants. [file 41073_2022_125_MOESM1_ESM.pdf]

Additional file 1.pdf [Online Supplement]

Jodi Schneider, Nathan D. Woods, Randi Proescholdt, and The RISRS Team. Reducing the Inadvertent Spread of Retracted Science: Recommendations from the RISRS Report. *Research Integrity & Peer Review*.

Table of Contents

Additional description of RISRS Workshops........2
Excerpt from survey before RISRS Workshop Day 3........3
Excerpt from survey after RISRS Workshop Day 3........7

Additional description of RISRS Workshops

In Workshop 1, participants were organized by stakeholder groupings and asked to reflect on some of the problems identified by the thematic analysis and to suggest additional problems associated with retraction that show up in their area of work or expertise. Problems under discussion included: types of retractions, informative retraction notices, the communication of misconduct investigations, visibility of retraction status, author-initiated retraction, the role of funding, the timeliness of retractions, and a variety of stakeholder nominated topics. In Workshop 2, stakeholders worked through problem modeling and structured deliberation to consider proposed long term and short term solutions to the continued citation of retracted research. Proposed solutions were drawn from the opportunities codes and themes from the interviews, and included: industry wide adoption of COPE guidelines; the use of retracted research evidence-based practice and policy; robust dissemination of retraction status; retraction education strategies; standards development processes; the development of end-user tools; and implementation of a widely adopted taxonomy of post-publication updates. Between Workshop 2 and 3, participants were surveyed on 7 salient recommendations derived from analysis of the prior workshops (see Excerpt from survey before RISRS Workshop Day 3, below). This provided stakeholders with an opportunity to rank recommendations and reformulate where necessary. Within those groupings they were further asked to rank components of those problems that held priority, or could be efficiently addressed. Finally, they were asked to nominate problems and opportunities not identified. During Workshop 3, the results of the survey were presented, culminating in 5 consensus recommendations, or areas stakeholders from the workshops agreed it would be productive to prioritize. For these 5 recommendations they were invited to again participate in small group discussion exercises to model possible implementation steps for each of the agreed upon recommendations. They were also invited to begin planning specific areas of collaboration leading out of the workshop, including in a post-workshop survey following Workshop 3 (see Excerpt from survey after RISRS Workshop Day 3, below).

Excerpt from survey before RISRS Workshop Day 3

RISRS Day 3 Agenda Setting

*** 1. Name**

2. Of the list of discussion groups topics for Day 3 which group would you like to join? Rank these in order of preference. (More info on these are below).

2. Of the list of discussion groups topics for Day 3 which group would you like to join? Rank these in order of preference. (More info on these are below).
A stand-alone, non-proprietary database to track and disseminate retraction status.
All stakeholders should adopt standard retraction metadata.
Develop a taxonomy of retraction statuses shared and adopted by all stakeholders.
Responsible Conduct of Research (RCR) Education
Strategy & Ownership for standards and best practice development

3. Suggest an additional topic or give further comments on these options.

Draft recommendations

We need your help to refine our draft recommendations.

1. Make retraction status information easy to find and use.

2. A stand-alone, non-proprietary database to track and disseminate retraction status.

3. All stakeholders should adopt standard retraction metadata.

4. Develop a taxonomy of retraction statuses shared and adopted by all stakeholders

5. Develop best practices guidelines for journals and institutions in addressing image problems.

6. Cover retraction as part of Responsible Conduct of Research (RCR) Education.

7. Display information about reproducibility of and confidence in scientific results.

4. Please suggest or rephrase the above recommendations:

1. Make retraction status information easy to find and use.

2. A stand-alone, non-proprietary database to track and disseminate retraction status.

3. All stakeholders should adopt standard retraction metadata.

4. Develop a taxonomy of retraction statuses shared and adopted by all stakeholders

5. Develop best practices guidelines for journals and institutions in addressing image problems.

6. Cover retraction as part of Responsible Conduct of Research (RCR) Education.

7. Display information about reproducibility of and confidence in scientific results.

5. Which recommendations do you agree with?

1. Make retraction status information easy to find and use.
1a. Ensure public access to retraction notices.
1b. Develop user interface conventions for flagging retracted works.
2. A stand-alone, non-proprietary database to track and disseminate retraction status.
2a. A sustainable, non-proprietary open access database focused on retraction status.
2b. Provide APIs to enter data into and pull data from Retraction Watch, the largest retraction status database, which has multiple partner organizations.
3. All stakeholders should adopt standard retraction metadata.
3a. Human and machine-readable metadata.
3b. Clear event history of the publication status.
3c. Retraction metadata is included in the metadata in the publishing system, travels upstream/downstream to partner organizations.
3d. Add retraction status to automated citation generation in Citation Style Language.
3e. Each version or status of a publication, including its retraction, should get a new persistent identifier (PID).
4. Develop a taxonomy of retraction statuses shared and adopted by all stakeholders.
4a. Stewarded by some stakeholder organization or group of organizations.
4b. Initially simple and coarse-grained (e.g. retraction, expression of concern, removal, ...)
4c. Continuously adopted while in development.
4d. Flexibility to add refinement later through an agile, iterative development process.

4e. Engage researchers, publishers, and standards organizations on standard identification language to reduce stigma around retractions.
4f. Machine readable and machine actionable.
4g. Included in the metadata in the publishing system, travels upstream/downstream to partner organizations.
5. Develop best practice guidelines for journals and institutions in addressing image problems.
6. Cover retraction as part of Responsible Conduct of Research (RCR) Education
6a. Develop sustainable funding for developing best practices in research integrity education
6b. Develop resources for discussing retraction in RCR education (e.g. specific ethics case studies, video describing retraction for researchers, etc.)
7. Display information about reproducibility of and confidence in scientific results
7a. Display information about citation agreement and disagreement in article reference lists (e.g. scite badge)
7b. Develop badges for journals to analyze and advertise their compliance with reproducibility and transparency-related reporting standards
Comments?
Enter text

6. What additional recommendations should we make?

7. Which likely attendees would you like to be in conversation with on Day 3?

[checkboxes on list of participants and affiliations appeared here]

8. Please list what OTHER people should be put in conversation with each other?

9. Anything else?

Excerpt from survey after RISRS Workshop Day 3

RISRS 2020 Post Workshop Momentum Building

Thank you again for participating in the workshop, "Reducing the Inadvertent Spread of Retracted Science." Together we have built up enthusiasm and momentum to tackle a very complex issue. Please help us to identify next steps going forward.

1. **Name:**

2. What plans do you have to address the inadvertent spread of retracted papers? What concrete steps can you take to implement these plans in the next 6 months?

3. Is there anything the research team can do to support your next steps? What relationships, resources, or information would facilitate your work?

4. Did the workshop introduce or reconnect you to anyone?

- ○ Yes
- ○ No

5. Are there workshop participants you plan to be in contact with?

6. Anything else you would like to share?
